# Malaria vector populations across ecological zones in Guinea Conakry and Mali, West Africa

**DOI:** 10.1186/s12936-016-1242-5

**Published:** 2016-04-08

**Authors:** Boubacar Coulibaly, Raymond Kone, Mamadou S. Barry, Becky Emerson, Mamadou B. Coulibaly, Oumou Niare, Abdoul H. Beavogui, Sekou F. Traore, Kenneth D. Vernick, Michelle M. Riehle

**Affiliations:** Malaria Research and Training Centre, Faculty of Medicine and Dentistry, University of Mali, Bamako, Mali; Centre de Formation et de Recherche en Santé Rurale de Mafèrinyah, Conakry, Republic of Guinea; Department of Microbiology, University of Minnesota, Minneapolis, MN USA; Department of Parasites and Insect Vectors, Unit of Genetics and Genomics of Insect Vectors, Institut Pasteur, Paris, France; CNRS Unit of Hosts, Vectors and Pathogens (URA3012), Paris, France

## Abstract

**Background:**

Malaria remains a pervasive public health problem in sub-Saharan West Africa. Here mosquito vector populations were explored across four sites in Mali and the Republic of Guinea (Guinea Conakry). The study samples the major ecological zones of malaria-endemic regions in West Africa within a relatively small distance.

**Methods:**

Mosquito vectors were sampled from larval pools, adult indoor resting sites, and indoor and outdoor human-host seeking adults. Mosquitoes were collected at sites spanning 350 km that represented arid savannah, humid savannah, semi-forest and deep forest ecological zones, in areas where little was previously known about malaria vector populations. 1425 mosquito samples were analysed by molecular assays to determine species, genetic attributes, blood meal sources and *Plasmodium* infection status.

**Results:**

*Anopheles gambiae* and *Anopheles coluzzii* were the major anophelines represented in all collections across the ecological zones, with *A. coluzzii* predominant in the arid savannah and *A. gambiae* in the more humid sites. The use of multiple collection methodologies across the sampling sites allows assessment of potential collection bias of the different methods. The L1014F *kdr* insecticide resistance mutation (*kdr*-*w*) is found at high frequency across all study sites. This mutation appears to have swept almost to fixation, from low frequencies 6 years earlier, despite the absence of widespread insecticide use for vector control. Rates of human feeding are very high across ecological zones, with only small fractions of animal derived blood meals in the arid and humid savannah. About 30 % of freshly blood-fed mosquitoes were positive for *Plasmodium falciparum* presence, while the rate of mosquitoes with established infections was an order of magnitude lower.

**Conclusions:**

The study represents detailed vector characterization from an understudied area in West Africa with endemic malaria transmission. The deep forest study site includes the epicenter of the 2014 Ebola virus epidemic. With new malaria control interventions planned in Guinea, these data provide a baseline measure and an opportunity to assess the outcome of future interventions.

**Electronic supplementary material:**

The online version of this article (doi:10.1186/s12936-016-1242-5) contains supplementary material, which is available to authorized users.

## Background

Malaria remains a prominent public health and financial burden across much of sub-Saharan African. To date, control efforts have been centered on vector control, largely through use of insecticide-treated bed nets (ITNs) or indoor residual insecticide spraying (IRS) [1]. Recent work has focused on potential genetic modification of vectors, but tools for practical implementation are not yet available. While insecticides are effective in the short term, selection of genetic resistance in the vector population by various mechanisms can decrease efficacy of control, or even allow rebound of transmission [2, 3].

Malaria intervention histories vary widely across Africa, and the Republic of Guinea (Guinea Conakry) has been relatively devoid of large-scale national malaria control efforts. The country has the eighth highest malaria mortality among 43 African nations, and the lowest proportion of children under 5 years old sleeping under an ITN, 47 %, as compared to 87 % in neighbouring Mali [4]. Guinea was the presumed site of emergence of Ebola virus from an animal reservoir that led to the 2014 West African epidemic [5, 6]. Malaria control and surveillance were neglected during the Ebola epidemic, and resurgence of malaria transmission and other public health problems are expected as a result [7–9].

There are also disparities in the level of knowledge of the malaria vectorial system between countries. While vector populations have been extensively studied in some areas of West Africa, for example Mali and Burkina Faso, information from Guinea Conakry is surprisingly sparse. Only one previous published paper characterizes mosquito vector populations in Guinea Conakry [10]. Nevertheless, the region offers unique opportunities, because the high ecological diversity in close proximity allows studies that capture all of the major ecological zones of malaria transmission across West Africa, while minimizing geographic variables. Moreover, because of the relative absence of prior national malaria control interventions, such as mass ITN distribution in Guinea Conakry, collected data can provide crucial baseline information on vector populations prior to intervention, a piece of data often missing in evaluation and planning of mosquito control strategies. In the current study, sampling was carried out across ecological zones from arid savannah in the north to deep forest in the south, and employed multiple collection methods at all sites. The analysis of the samples characterizes the vector population in a little-studied region of West Africa.

## Methods

### Mosquito collections

**Fig. 1:**
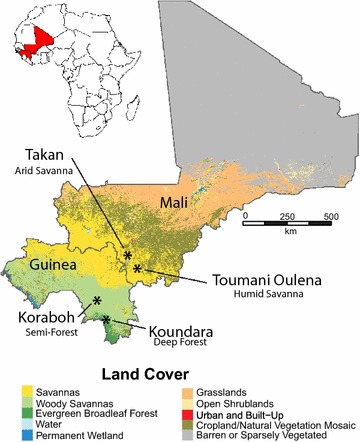
Study sites and ecological zones. Four study sites were sampled, including the savannah villages of Takan and Toumani Oulena just north of the border with Mali, an intermediate semi-forest site in the village of Koraboh, and the deep forest in the village of Koundara on the border with Liberia. *Map* is *shaded* for land cover type with data from [41]. The locations of Guinea Conakry and Mali are indicated in *red* on the *inset map* of Africa

Mosquitoes were collected from four different study sites at the border with Mali and in Guinea Conakry (Fig. 1). Takan (N 11.47, W 8.33) and Toumani Oulena (N 10.83, W 7.81) are both small villages in the Yanfolila district of southern Mali and represent the Sudanian savannah ecological zone. Takan is arid savannah, while Toumani Oulena is humid savannah. In Guinea Conakry, we sampled in Koraboh, (N 9.28, W 10.03) a small village in the Kissidougou district in the Faranah region representing a semi-forest site with intermediate ecology, a mix of savannah and forest, and in Koundara, (N 8.48, W 9.53), a small village in the Macenta district in the Zezekore region representing deep forest ecology. All molecular attribute data for mosquitoes is available (Additional file 1).

All reported collections occurred in October and November in 2012. In the Yanfolila district of Mali, which includes Takan and Toumani Oulena study sites, the overall mean temperature range was 21–30 °C and the mean relative humidity range is 37–73 %, although Takan is more arid and Toumani Oulena more humid. The average temperature at the Koundara site was 21 °C with 92 % relative humidity while in Koraboh it was 24 °C and 86 % relative humidity. Epidemiological data for malaria are sparse, although self-presented cases of fever at district-level clinics from 2006 (uncorrected for district population size) can provide an estimate [11]. The savannah sites displayed a highly seasonal pattern with a peak of >2000 fever cases/month from June–September and very low otherwise, similar to the well-studied Sudanian savannah zones of neighbouring Mali or Burkina Faso [12–14]. The district of Kissidougou, including the semi-forest site of Koraboh, displayed >2500 cases/month in every month except April. The district of Macenta, including the deep forest site of Koundara, displayed >1400 cases/month in every month of the year, with a peak of 1700 in September and October.

At each site, mosquitoes were collected using three different methods: (1) human-landing capture (HLC), (2) indoor manual aspirator or pyrethroid spray catch, and (3) larval capture, where the first and second instar larvae were raised to adult in a field insectary under standard insectary conditions prior to DNA isolation from the adults; and the third and fourth instar larvae were preserved directly for DNA isolation, without rearing in the insectary. The two distinct methods of larval collection were used to control for possible genetic bias inherent in lab rearing of captured larvae. Across sites, all types of larval sites were sampled, including both temporary and permanent sites. Human-landing captures were performed both inside dwellings and outside (>10 m from dwelling) at night between 18:00 and 06:30. The indoor aspirator or spray catches were done in the morning between 06:00 and 12:00. Adult specimens or third and fourth instar larvae were preserved immediately in 80 % ethanol until later DNA extraction. First and second instar larvae were raised to adults in nearby field insectaries and upon emergence were preserved in 80 % ethanol. DNA was extracted from mosquitoes using DNAzol by the provided protocol (Invitrogen, CA, USA).

### DNA isolation and species identification

Genomic DNA was isolated from individual mosquitoes using DNAzol (Invitrogen, CA, USA). DNAs were resuspended in distilled water and stored at −20 °C. All samples were typed by a molecular diagnostic assay to determine species status within the *A. gambiae* species complex [15]. If this assay failed to yield a diagnostic band, the ribosomal gene ITS2 region was PCR amplified [16], Sanger-sequenced, and the resulting sequence was used to search the NCBI nr database using blast. Mosquito species calls based on ITS2 sequence used a threshold of >98 % nucleotide identity. In total, 390 samples were analysed from Koraboh, 329 from Koundara, 345 from Takan and 361 from Toumani Oulena. All samples were genotyped for mosquito species. For the remainder of attributes, all human-landing capture samples and random subsets of spray and larval caught samples were analysed, with sample sizes indicated with each figure. All genomic DNAs were isolated from whole mosquitoes or larvae.

### Genotyping the 2La chromosome inversion

Molecular karyotyping of the paracentric 2La inversion was done using published methods [17]. When product bands other than those reported were seen, they were sequenced and compared with other non-standard product sizes previously reported [18, 19]. These non-standard sizes were assigned as wild-type (2La^+^) or inverted (2La) based on sequence and published information. The non-standard sizes are generally the result of insertion of mobile elements such as MITEs into the inversion breakpoint [19].

### Genotyping of the *kdr*-*w* mutation

The mutation of the para gene, L1014F, called West African *kdr* or *kdr*-*w*, which is associated with pyrethroid insecticide resistance, was genotyped by a published PCR diagnostic assay [20], and in a small number of samples (n = 15–18 samples per population) a short amplicon generated with the Agd1 and Agd2 primers was amplified and sequenced to genotype the East African *kdr* mutation, L1014S, also called *kdr*-*e*. The geographic names of these mutations are a misnomer, as the East African *kdr* mutation has been detected in samples from West Africa [21]. Across all sampling sites, *kdr* genotypes for 70 samples were confirmed by Sanger sequencing.

### Molecular detection of blood meal source

To determine the source of blood meal, published assays were modified based on amplification of the mitochondrial *cytB* gene. Blood meal source was typed in all those mosquitoes with a visibly detectable blood meal. Visible blood meal rates were >95 % in indoor spray-caught samples and also included some human-landing capture samples, presumably seeking a second blood meal in the same gonotrophic cycle. All samples were initially typed with a published method [21], but due to amplification of mosquito DNA in negative control samples, a new reverse primer was created and optimized to minimize mosquito amplification. Using cytb forward 5′-GAG GMC AAA TAT CAT TCT GAG G-3′ from [21] and newly designed cytb Rev 5′-CGR AAT ATT ATG CTT YGT TG-3′ samples were assayed in a 25 µl PCR reaction containing 1.5 µl undiluted genomic DNA, 1 × PCR buffer, 3 mM MgCl2, 0.4 µM primers. Cycling conditions were an initial denaturation of 95 °C for 1 min followed by 35 cycles of 95 °C for 30 s, 56 °C for 50 s and 72 °C for 1 min followed by a final extension of 72 °C for 5 min.

The primers amplify a 549 bp fragment that was subsequently digested with restriction enzymes, *Ear*I and *Apa*I. *Ear*I is diagnostic for blood meals derived from humans and goat/sheep. If the blood meal is human in origin, the *Ear*I digest cuts once resulting in bands of 248 and 301 base pairs. If the blood meal is goat or sheep in origin, the band sizes following *Ear*I digestion will be 194 and 355 base pairs. Distinguishing goat from sheep requires sequencing of the original band. Digestion of the *cytB* amplicon with *Apa*I yields products 257 and 292 bp in length if the blood meal is bovine in origin. All products that are uncut after independent digestions with *Ear*I and *Apa*I were Sanger sequenced and blasted against the nr database at Genbank. All blood meal assays included positive control animal genomic DNAs purchased from a commercial source (Zyagen, CA, USA). A reconstruction experiment was done by serial dilution of human DNA into mosquito DNA, which determined that within a typical mosquito DNA sample, the limit of detection for human DNA was 0.05–0.005 pg.

### Molecular detection of *Plasmodium falciparum* in blood-fed and unfed mosquitoes

The presence of *Plasmodium falciparum* was determined using a PCR assay that amplifies a portion of the parasite *cytB* gene. The assay is a modification of reported methods [22, 23], producing a 902 bp PCR fragment. Initially the assays were run independently and were later combined and modified to improve specificity and sensitivity. The PCR based detection of *P. falciparum* used forward primer PFcytblongF 5′-ATACATGCACGCAA CAGGTGCTTCTC-3′ [22] and reverse primer MitR2 5′-TGTTTGCTTGGGAGCTGTAA-3′ [23]. The PCR reaction was 1x Accuprime Supermix II (Invitrogen, CA, USA), 1 pmol of each primer and 2 µl of undiluted mosquito genomic DNA (or 1 µl of control *P. falciparum* DNA serial dilution). PCR conditions were 35 cycles at 94 °C for 40 s, 61 °C for 40 s and 72 °C for 90 s. The limit of detection was determined by serial dilution of *P. falciparum* DNA, ranging from 6.775–0.0006775 pg, and a standard curve was run with every PCR. Serial dilutions of *P. falciparum* stock DNA were made fresh for each independent PCR experiment, and control reactions were run alongside all experimental samples. The limit of detection for *P. falciparum* was 0.06775 pg/µl (or ~2 *P. falciparum* genome equivalents). All samples that tested positive were independently retested to confirm results.

The *P. falciparum* detection assay was performed on all adult mosquito samples positive for a human blood meal (i.e., visually positive for a blood meal and positive for human blood) to estimate the *P. falciparum* exposure rate (the rate at which mosquitoes take a blood meal that contains *P. falciparum* parasites). The *P. falciparum* assay was also performed on all adult samples not positive for a visible blood meal, to estimate the rate of established infections in the mosquito. Exposure rate was estimated from all blood-fed mosquitoes, including indoor-resting spray catch as well as HLC.

### Ethical considerations

For human-landing capture of mosquitoes, the study protocol was reviewed and approved by the institutional and national health ethical review boards (Comité National d’Ethique pour la Recherche en Santé) of The Republic of Guinea (reference number N′003/CNFRSR/MAF/13) and (Comité d’Ethique Institutionelle/FMPOS) of the Republic of Mali (reference number Traore-37). The study procedures, benefits and risks were explained to collectors and their informed consent was obtained. All collectors were followed and symptomatic subjects were treated with the combination of artemether–lumefantrine (Coartem^®^) according to relevant regulations of the Republic of Guinea and Republic of Mali Ministries of Health.

## Results and discussion

A 350 km ecological sampling zone was established from the Mali border in the north of Guinea Conakry, to the south of the country near the border with Liberia (Fig. 1). Study sites were located in the following ecological zones: arid savannah (village of Takan), humid savannah (Toumani Oulena), semi-forest (Koraboh), and deep forest (Koundara). The range of ecologies sampled across this relatively short distance is representative of the major zones of malaria transmission present throughout West Africa.

Adult mosquitoes were sampled by techniques that collect behaviourally discrete groups of mosquitoes: human-landing capture (HLC) inside and outside of houses (indoor and outdoor human host-seeking), and pyrethroid spray capture inside houses (indoor-resting). In addition, larvae were sampled from breeding pools around the villages, which should represent an unbiased collection of *Anopheles* species in proportion to their relative abundance in the overall population. This study surveys mosquito vectors in Guinea Conakry and Mali for species composition, blood meal source, and multiple molecular attributes with potential importance to vector control and malaria transmission. The data permits comparison of the different features according to ecological variables, as well as comparison with the only other reported survey of Guinea Conakry vectors, using mosquitoes collected 6 years prior in 2006 [10].

### Species composition

**Fig. 2:**
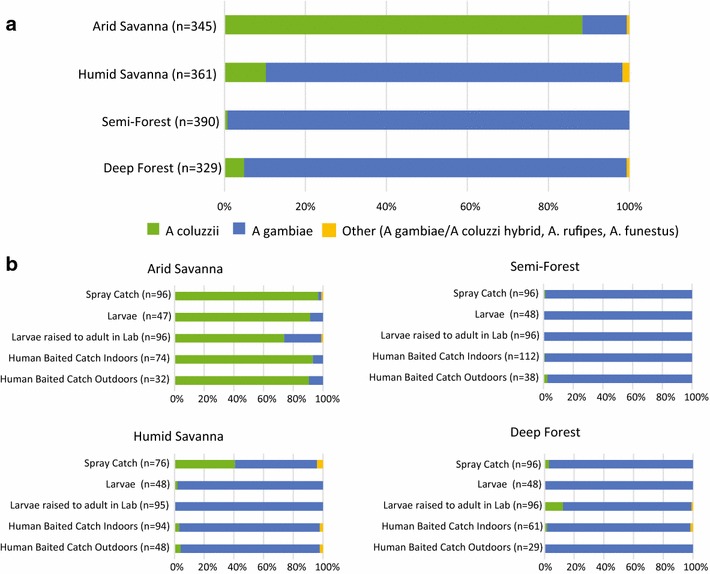
Mosquito species composition across the ecological zones. *Anopheles coluzzii* is predominant in the arid savannah, while *A. gambiae* predominates in the humid savannah, semi-forest and deep forest sites. **a** Species composition across the ecological zones. **b** Species sampled per study site by the different collection methods

At all sites, the vast majority of mosquito samples were comprised of the sister taxa, *A. gambiae* (formerly S molecular form) and *A. coluzzii* (formerly M molecular form). *Anopheles gambiae* predominated in the semi-forest and deep forest sites while in the two savannah sites both species were present, with *A. coluzzii* predominant in the arid savannah and *A. gambiae* in the humid savannah (Fig. 2). The different sampling strategies were generally consistent for the species captured. In the savannah locations where both species were present, the predominant species present was sampled equivalently across sampling methods, whereas the minor species fluctuated by method. *Anopheles coluzzii* in the humid savannah was most represented among the spray caught samples, and *A. gambiae* in the arid savannah was most represented among larval collections (Fig. 2). This suggests that the spatial distribution of the non-predominant species may be less widespread and patchier at a given site, and could potentially be missed using only a single sampling method. In the arid savannah, the higher frequency of *A. gambiae* among larvae raised to adult in the lab suggests that the controlled lab conditions may remove selection pressures against *A. gambiae* that influence survival in the natural larval pool. The species composition of the larval samples was similar to the wild-captured adults (spray and HLC), further suggesting that larvae raised to adults in the laboratory altered the emergence success of *A. gambiae* larvae (Fig. 2b). Despite the difference in predominant vector species across sites, it has previously been demonstrated that *A. coluzzii* and *A. gambiae* do not differ in their susceptibility to *Plasmodium* or in their role in transmission [24–27]. Consequently, the difference in species composition across sites is not expected to have direct impact upon malaria transmission.

Finally, a small number of other species were found, including the vector *Anopheles funestus*, the largely zoophilic *Anopheles rufipes*, and *A. gambiae/A. coluzzii* hybrids. Interestingly, no *Anopheles arabiensis* were sampled among the 1425 mosquitoes across all sampling methodologies and ecologies. This contrasts with species present in the Sudanian savannah zone of Burkina Faso, also during the rainy season as in the current study, where *A. arabiensis* comprised nearly 50 % of the larval pool species composition [24].

In the previous reported survey in Guinea Conakry based on collections made in 2006 [10], two sites displayed predominantly *A. gambiae*, with *A. coluzzii* present at >5 %. One of these sites, Siguiri, is ecologically comparable to the current semi-forest site of Koraboh with similar species composition, while the other site, Boffa, is in the west of Guinea Conakry, a region not included in the current study. However, at Siguiri 1.3 % (2/156) *A. arabiensis* were reported previously, while none were observed in the current study using twice the sample size. The third site of the 2006 sampling was Mt Nimba, in the south near the current deep forest site of Koundara. Mt Nimba displayed 38 % *A. gambiae* and 61 % *A. coluzzii*, in distinction to the predominance of *A. gambiae* at Koundara. However, the Mt Nimba site appears to be a more arid disturbed habitat as compared to Koundara (Fig. 1 compared to [10]), thus not ecologically equivalent, and probably more suitable for *A. coluzzii*. In 2006, *A. funestus* were also present (Siguiri n = 15, Mt Nimba n = 35) while in the current study only one was collected, at the humid savannah site. The 2006 samples were collected in July–August and the current samples in October–November, so there could be a seasonal difference. Overall, the species distributions appear largely similar between the 2006 and 2012 collections.

## 2La inversion genotype

**Fig. 3:**
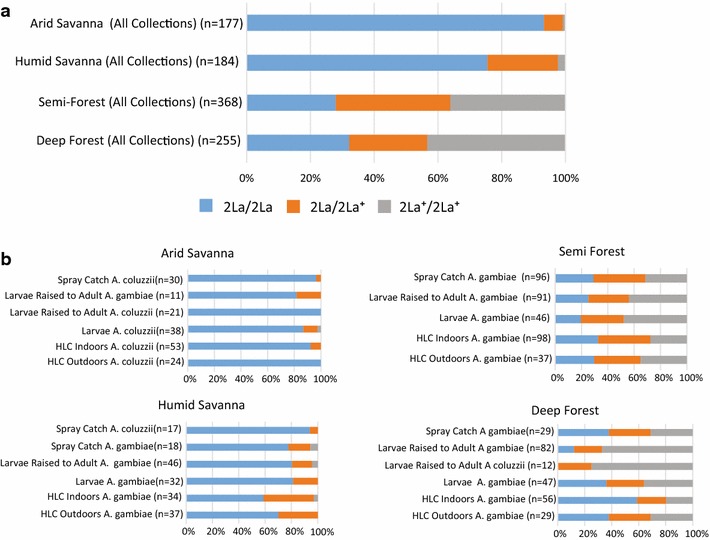
Distribution of the paracentric 2La chromosome inversion. **a** The wild-type 2La^+^ allele is more prevalent in forest ecologies as compared to savannah ecologies. The frequency of 2La^+^ alleles appears to follow an ecological cline. **b** Rates of 2La alleles per study site by collection methodology. In the arid and humid savannah sites where the comparison can be made, *A. coluzzii* carried fewer 2La^+^ chromosomes than sympatric *A. gambiae* collected by the same sampling methodology

The 2La paracentric chromosome inversion was genotyped because this large feature, comprising ~10 % of the genome, is correlated with aspects of vector bionomics, including genotype frequency association with mosquito resting behavior and ecological aridity [28, 29]. Consistent with those observations, in Guinea Conakry and Mali the frequency of the aridity-associated inverted allele (2La) was highest in the arid savannah site at 96 %, followed by 86 % in the humid savannah (Fig. 3). The rate of the 2La allele declines to 46 % in the semi-forest and 43 % in the deep forest. The frequencies of 2La and 2La^+^ alleles vary significantly across the ecological zones (Chi Square = 400.487, p > 10e−10) and vary significantly across all pairs of sites, expect the deep forest and semi-forest which are not significantly different (Chi Square = 0.233, p = 0.63). 2La inversion frequencies are generally consistent across sampling methodologies, although there can be subtle differences. For example, larvae raised to adults in the insectary carried fewer inverted 2La alleles than other collection methods, including direct larval collections, suggesting that laboratory conditions may alter the efficiency of larval survival or adult emergence. The similarity between larval collected samples and natural adult collections (HLC and spray catch) suggests that adult emergence is not different from larval composition (Fig. 3b). In addition, the humid savannah samples captured seeking a human blood meal carried a higher frequency of the non-inverted 2La^+^ allele than mosquitoes collected by other methodologies (18 versus 10 %).

### *kdr* mutation frequency

**Fig. 4:**
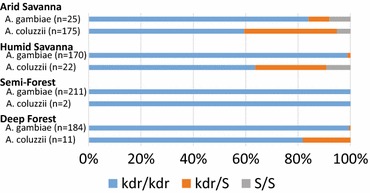
The *kdr*-*w* mutation associated with insecticide resistance is nearly fixed in the vector population. The *kdr*-*w* mutation, L1014F, in the *para* sodium ion channel gene is prevalent across all sites. Samples from deep forest and semi-forest sites are essentially fixed for *kdr*-*w*, while the wild-type allele still segregates in the arid and humid savannah. Consistent with directional introgression of a *kdr*-carrying haplotype from *A. gambiae* into *A. coluzzii*, frequency of the *kdr*-*w* allele was higher across sites in *A. gambiae* (0.99 in *A. gambiae* and 0.77 in *A. coluzzii*)

Mutations in the *para* voltage gated sodium ion channel gene (AGAP004707), collectively referred to as knockdown-resistance or *kdr*, are associated with resistance to pyrethroids [20, 30]. Pyrethroids are the only approved insecticide for ITN use [31, 32], presenting a challenge in malaria vector management. Guinea Conakry was subject to little widespread malaria control effort prior to the current sampling in 2012, while in neighboring Mali far more extensive malaria control campaigns using IRS and ITNs have occurred [4]. Despite the difference in malaria control regimes, the frequency of the L1014F *kdr*-*w* mutation is high across all study sites (0.77–1.0), and is fixed in all samples assayed from the semi-forest site (Fig. 4). All three *kdr*-*w* genotypes segregate in *A. coluzzii* at the arid savannah site, yielding the power to test for bias or differential collection efficiency by sampling method. No difference was detected in genotype frequency across the different methods of collection (Chi Square = 3.31, df = 6, p = 0.77). However, the rates of the *kdr*-*w* insecticide resistance allele are significantly lower in *A. coluzzii* as compared to *A. gambiae* (21.9 % allele frequency in *A. coluzzii* as compared to 99 % in *A. gambiae*, Chi Square = 230.55, df = 1, p < 0.0001). This observation is consistent with literature describing introgression of the *kdr* mutation from *A. gambiae* into *A. coluzzii* [33–37]. The L1014S mutation was also examined in a subset of samples. L1014S, also called *kdr*-*e* for East Africa but known to occur in West Africa, was not detected in 15–18 samples per site. In a subset of 70 samples, *kdr* genotype calls were verified by manual Sanger sequencing, and in all cases sequence results were concordant with the result of the PCR diagnostic assay.

It is interesting that the previous reported survey in Guinea Conakry based on collections made in 2006 displayed considerably lower *kdr* frequencies [10]. In 2006, the L1014F *kdr*-*w* allele was present in *A. gambiae* at a frequency of 0.14 from Mt. Nimba, near the current deep forest site that displayed *kdr*-*w* frequency of 0.997 in *A. gambiae* 6 years later in 2012 (of 384 mosquitoes, only one was heterozygous). The 2006 collection detected the *kdr*-*w* allele present at a frequency of 0.24 in *A. gambiae* at a site in the west of the country that is not comparable to any of current sites. The 2006 overall *kdr*-*w* allele frequency in *A. coluzzii* was 0.03, while by 2012 the overall frequency in *A. coluzzii* was 0.77 and *A. gambiae* was 0.99. In 2005, the proportion of households with at least one ITN was <1 % while by 2012 the figure had increased to 47 %, even in the absence of previous national mass distribution of ITNs, which was planned to begin in 2013 or 2014 [4]. The observed *kdr* increase could thus be a consequence of exposure to ITNs in the intervening 6 years [36], or may be driven by the agricultural use of pesticides, or by other ecological changes. In any case, from 2006 to 2012, although vector species composition remained essentially stable, the frequency of *kdr*-*w* increased sharply. By 2012, before the first national mass ITN distribution in Guinea Conakry, *kdr*-*w* was already essentially fixed in the vector population.

### Bloodmeal source

**Fig. 5:**
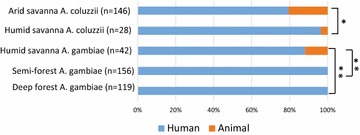
Human blood meals predominate across ecological sites. Consistent with being highly anthropophilic, *A. gambiae* and *A. coluzzii* samples carrying visually detectable blood meals most often contain human blood meals across geographic study sites. Mosquitoes from the arid and humid savannah display a proportion of animal blood meals, while semi-forest and deep forest mosquitoes appear to be entirely human-feeding. *Anopheles coluzzii* captured in the arid savannah are more likely to have consumed an animal blood meal than *A. coluzzii* from the humid savannah (Fisher’s exact test, p = 0.03). *Anopheles gambiae* captured in the humid savanna were significantly more likely to contain animal blood meals than *A. gambiae* from the semi- and deep forest zones (Fisher’s exact test, p < 0.001). Animal blood meals were derived from cows, dogs, donkeys, and sheep. *Graph* labels, *p < 0.05, **p < 0.001

In the deep forest and semi-forest sites, all detectable mosquito blood meals were of human origin (Fig. 5). In the savannah sites, more animal blood meals were observed. *A. coluzzii* carried significantly more animal blood meals in the arid savannah than the humid savannah site (p = 0.03). The animal blood meals originated from cows, dogs, donkeys and sheep. The *A. gambiae* animal feeding rate at the humid savannah site, where there were adequate numbers to compare, was not distinguishable from *A. coluzzii* (p = 0.39). In a comparison of *A. gambiae* across sites, animal-derived blood meals were significantly more likely in the humid savannah site than in either the deep forest or semi-forest (p < 0.001). The predominance of human derived blood meals observed across ecological zones is similar to published results from other West and Central African countries, although previous studies did not examine the range of ecologies sampled here [27, 38, 39]. Animal blood meals have been shown to be more common when humans are less accessible, potentially due to use of ITNs [40]. The current study represents the first report for Guinea, as the previous work did not report on blood meal source [10].

### *Plasmodium* presence

The presence of *P. falciparum* was measured among the mosquitoes carrying a human blood meal, and was termed the exposure rate, that is, the rate at which mosquitoes taking a human blood meal were exposed to parasites. Exposure rate includes the parasite challenge rate by infectious gametocytes, but it also includes other unknown factors: the developmental stage of parasites, including maturity of gametocytes if any, as well as the rate of signals originating in previously established infections and not the blood meal. *Plasmodium falciparum* presence was also measured in mosquitoes not carrying a visible blood meal, and was termed the rate of established *P. falciparum* infections since the signal could not have originated directly from blood. Partitioning exposure and established infection was done to avoid bias and to obtain the most robust measures of these two epidemiologically important values. Exposure rate was estimated from all blood-fed mosquitoes, including indoor-resting spray catch as well as HLC.

**Fig. 6:**
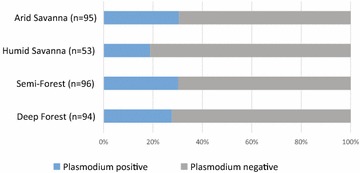
Mosquito exposure to *Plasmodium falciparum* was similar across sites. All mosquitoes positive for a human blood meal were screened for presence of *P. falciparum* to estimate the parasite exposure rate, which was 20–30 % at all sites

The *P. falciparum* exposure rate among indoor spray-caught individuals did not differ among study sites (Chi Square = 2.736, df = 3, p = 0.434), and averaged between 20–30 % of mosquitoes positive for a human blood meal (Fig. 6; in deep forest 26/94 tested positive for *P. falciparum* exposure, in semi-forest 29/96, in humid savannah 10/53 and in arid savannah 29/95). The rates of *P. falciparum* established infection, measured in mosquitoes not carrying a detectable blood meal, were statistically indistinguishable between study sites (Chi Square = 2.457, df = 3, p = 0.483). The rate of established infection in the humid savannah was 3.3 % (3/92). No established infections were detected at the other ecological sites, but the difference from the humid savannah is not meaningful because the sample sizes of human blood-fed mosquitoes lacks statistical power (n = 28 in the arid savannah, 12 in the deep forest and 34 in the semi-forest). The established infection rate is likely to be an underestimate because the true challenge rate of the tested mosquitoes is unknown, for example, the fraction of non blood-carrying mosquitoes that were recently emerged and had not previously fed.

## Conclusions

Mosquito sampling and analysis throughout four ecological zones spanning the range of malaria transmission ecologies present across West Africa provides useful insights for the design, implementation and evaluation of vector control strategies. Additionally, choice of vector sampling method can influence the conclusions that are reached. Baseline data presented here, collected prior to systematic vector control intervention, can be valuable to design, evaluate and monitor vector control strategies. Given that 25–34 % of human host-seeking (HLC) mosquitoes were collected outdoors, indoor-directed control measures (e.g., ITNs and IRS) will miss a portion of the vector population and thus may have limited effectiveness to control vectors. Despite the low level of previous mass distribution of ITNs in Guinea Conakry, the vector population is nearly fixed for the *kdr*-*w* resistance mutation. This could diminish both immediate and long-term impact of ITNs for malaria control in the country.

## Additional file

10.1186/s12936-016-1242-5 Molecular attribute data for mosquitoes collected across ecological zones in Guinea and Mali.
